# Pediatric eosinophilic esophagitis: a review for the clinician

**DOI:** 10.1186/s13052-021-01178-2

**Published:** 2021-11-22

**Authors:** Simona Barni, Stefania Arasi, Carla Mastrorilli, Luca Pecoraro, Mattia Giovannini, Francesca Mori, Lucia Liotti, Francesca Saretta, Riccardo Castagnoli, Lucia Caminiti, Antonella Cianferoni, Elio Novembre

**Affiliations:** 1 Allergy Unit, Department of Pediatrics, Meyer Children’s University Hospital, Florence, Italy; 2 Predictive and Preventive Medicine Research Unit, Multifactorial and Systemic Diseases Research Area, Pediatric Allergy Unit, Bambino Gesù Children’s Hospital IRCCS, Rome, Italy; 3 Pediatric Unit and Emergency, University Hospital Consortium Corporation Polyclinic of Bari, Pediatric Hospital Giovanni XXIII, Bari, Italy; 4 Department of Medicine and Surgery, University of Parma, Parma, Italy; 5 Department of Medicine, University of Verona, Policlinico GB Rossi, Verona, Italy; 6 Pediatric Unit, ASST Mantua, Mantua, Italy; 7 Pediatric Unit, Senigallia Hospital, Senigallia, Italy; 8 Pediatric Department, Latisana-Palmanova Hospital, Azienda Sanitaria Universitaria Friuli Centrale, Udine, Italy; 9 Department of Pediatrics, Pediatric Clinic, Fondazione IRCCS Policlinico San Matteo, University of Pavia, Pavia, Italy; 10 Department of Human Pathology in Adult and Development Age “Gaetano Barresi”, Allergy Unit, Department of Pediatrics, AOU Policlinico Gaetano Martino, Messina, Italy; 11 Pediatrics Department, Perelman School of Medicine, University of Pennsylvania, Philadelphia, USA; 12 Allergy and Immunology Division, The Children’s Hospital of Philadelphia, Philadelphia, USA

## Abstract

Eosinophilic esophagitis (EoE) is a chronic clinical-pathologic disease characterized by eosinophilic infiltration of the esophageal epithelium with esophageal dysfunction symptoms.

EoE can occur at any age and has different clinical manifestations depending on the age onset.

To date, esophago-gastroduodenal endoscopy (EGD) with biopsy is the gold-standard for EoE diagnosis.

According to the recent consensus guidelines, proton pump inhibitors, corticosteroids and elimination diets could be a first-line therapy option. The aim of the treatment is clinical and histological remission for preventing long-lasting untreatable fibrosis.

A multidisciplinary approach (allergist, gastroenterology, dietitian, and pathologist) is recommended for managing patients affected by EoE, given the complexity of its treatment.

This review will provide a practical guide to assist pediatricians treating children with EoE.

Moreover, it highlights the unmet needs in diagnosis and treatment that require urgent attention from the scientific community in the aim of improving the management of patients with EoE.

## Introduction

Eosinophilic esophagitis (EoE) is a chronic immune-mediated disease characterized by clinical manifestations related to esophageal dysfunction and, histologically, by esophageal eosinophilic inflammation [1].

In recent decades, the incidence and prevalence of EoE has increased [2–4], being the leading cause of dysphagia in children and young adults [5]. Currently, esophageal biopsy through upper gastrointestinal endoscopy still remains the gold standard diagnostic test to perform when EoE is suspected [1]. Since upper endoscopy is an invasive and expensive procedure, less invasive tools are being developed to evaluate esophagus inflammation, however, larger studies are needed to validate these tests [6–12].

According to the last consensus guidelines, proton pump inhibitors (PPIs) are considered first-line therapeutic options on the same level as steroids and elimination diet [1]. The treatment must be individualized according to each patient’s lifestyle and family situation and could be interchangeable over time [13].

The aim of this review is to update the current evidence on EoE in children, moreover, it is intended as a practical guide for clinicians treating patients with EoE in order to avoid delayed diagnosis which can lead to inflammatory progression and ultimately, fibrostenosis [14].

## Research strategies and literature analysis

We reviewed the most relevant studies on “Pediatric Eosinophilic Esophagitis” present in databases including PubMed (https://www.ncbi.nlm.nih.gov/pubmed/) and the Cochrane Library up until 2020. Manuscripts were selected from randomized controlled trials, case reports, reviews, systematic reviews, cohort and case–control studies, and observational studies. Single-case reports, abstracts/posters without sufficient detail, articles with duplicated data, and articles in non-English language were all excluded. The terms searched for were “pediatric eosinophilic esophagitis” [all fields]; “eosinophilic esophagitis” and “children” [all fields]; “pediatric eosinophilic esophagitis” and “management” [all fields]; “pediatric eosinophilic esophagitis” and “treatment” [all fields]; “pediatric eosinophilic esophagitis” and “therapy” [all fields]; “pediatric eosinophilic esophagitis” and “diet” [all fields]; “esophageal eosinophilia” [all fields]; “pediatric GERD” [all fields]; “gastroesophageal reflux” and “children” [all fields].

## Epidemiology

EoE has been characterized as a disease relatively recently, with the first description by Landres et al. in 1978 [15]. Over time, EoE has gained more and more interest and, in parallel, the epidemiology of EoE has also rapidly increased, in particular after the publication of the consensus recommendations for diagnosis and management of the disease in 2007 [16]. However, the increase in recognition and knowledge of EoE would not seem to be the only explanation for such a rise in frequency [1]. This observation might entail implications for the knowledge of the etiology of EoE which has transformed from a rare case-reportable condition into a major cause of food impaction and the leading cause of dysphagia in children and young adults [5].

Most data come from population-based studies, conducted primarily in North America and Europe [2, 3, 17–21]. Incidence rates range from 2.1/100,000/year in the Netherlands [4] to 12.8/100,000/year in Ohio in the United States [22]. In 2016, a meta-analysis evaluated the epidemiology of EoE reported by various countries and calculated an overall pooled incidence of 3.7/100,000/year (95% confidence interval [CI], 1.7–6.5) which was higher in adults (7/100,000/year; 95% CI:1–18.3) than in children (5/100,000/year; 95% CI: 1.5–10.9). The same study assessed an overall prevalence of 22.7 cases per 100,000 inhabitants, being the highest in North America with a prevalence of 30.7 cases per 100,000 inhabitants, despite substantial heterogeneity [23].

However, when interpreting the published epidemiology data, it is important to recognize heterogeneities among studies performed at different centers and during different periods. The reported frequency varied widely, probably due to differences in multiple factors such as clinical and laboratory criteria, patients’ ages, duration of illness, and geographic variations in dietary habits. Further data from well-designed homogeneous studies are necessary.

## Pathogenesis

EoE is characterized by a multifactorial esophageal inflammation occurring in individuals with genetic predisposition, which combines an esophageal epithelia dysfunction and an abnormal T-helper cell type 2 (Th2)-mediated immune response to environmental allergens, and leads to esophageal lesion and dysmotility, secondary remodeling and fibrosis [24–26].

### Genetic factors

The risk of developing EoE is higher among first-degree family members, in particular, being a father or a brother of a patients affected by EoE, increases the risk of developing the disease 43-fold and 64-fold respectively, whereas the EoE frequency exhibited in monozygotic and dizygotic twins is 41 and 22% respectively [7].

Multiple genome-wide association studies (GWAS) have been conducted to identify genetic loci associated with EoE [27, 28]. Some of the genes have known functions, while others are still unknown [28]. Most of the genetic loci identified affect epithelial barrier functions and Th2-mediated immune responses [29, 30].

The thymic stromal lymphopoietin (TSLP) gene has been associated with EoE in multiple independent studies. TSLP is an epithelial cytokine that favors a pro-Th2 environment [27, 31, 32]. However other genes have emerged in the EoE pathogenesis that are located in the epidermal differentiation complex (1q21), several of which are dysregulated and involved in epithelial cell differentiation [33].

Desmoglein-1 (DSG1), downregulated by interleukin (IL)-13, gives rise to an epithelial cell barrier disfunction [31].

Calpain-14 (CALPN14), an esophagus-specific proteolytic enzyme induced by IL-13, leads to a loss of DSG1 expression and impaired epithelial barrier function [34]. Moreover, filaggrin (FLG), a fundamental epithelial protein, is downregulated in EoE [30].

Lastly, the serine peptidase inhibitor Kazal type 5 and 7 (SPINK5 and SPINK7) participates in EoE pathogenesis because the activity of serine proteases is not under control [30].

As a result, increased esophageal tissue permeability and antigen uptake could give rise to an abnormal Th2-immune response. This involves several cytokines such as IL-4, IL-5 and IL-13 [24, 25]. IL-4, secreted by Th2 cells, natural killer (NK) cells, and basophils, which induce the differentiation of T cell into Th2 and active B cells [25, 35]. IL-5, secreted by Th2 cells, mast cells, and eosinophils, promotes eosinophil proliferation, survival, activation, and chemotaxis [36–38].

In addition to the effects mentioned above, IL-13, secreted by Th2 cells, upregulates eotaxin-3 (CCL26) that causes chemotaxis of the eosinophils [33]. Moreover, IL-13 upregulates periostin (POSTN) and transforms the growth factor (TGF)-β, increasing the adhesion of the eosinophils to fibronectin [39]. Furthermore, TGF-β directly induces the expression of profibrotic genes, such as fibronectin, collagen I, periostin, and smooth muscle actin, causing esophageal epithelial fibrosis and decreasing esophageal smooth-muscle contraction [40, 41].

Ultimately, IL-13 induces tissue remodeling by promoting collagen deposition, angiogenesis, and epithelial hyperplasia [37, 42].

A close relationship between DSG1 and POSTN highlights the interaction between epithelial barrier dysfunction and the Th2-mediated immune response: when DSG1 is downregulated, POSTN increases, triggering the production of TSLP by the esophageal epithelium. TSLP induces a Th2-immune response and is implicated in eosinophil survival, and mast cell and basophil activation [40, 43–45].

STAT6 (12q13), which is associated with the pathogenesis of EoE. is activated by IL-4 and IL-13 and encodes for a transcription factor activating many EoE genes [29, 30].

### Environmental factors

The rapid increase in the incidence and prevalence of EoE demonstrates the paramount role of environmental factors in its pathogenesis [24, 26, 46]. The exposure to environmental allergens can also play a critical role in developing the disease and its exacerbation, such as the pollen season, Fall, or living in a cold or dry climate [24].

Moreover, living in a rural environment increases the risk of developing EoE because of increased exposure to aeroallergens [47]. Furthermore, animal models support a link between antigen exposure and EoE [48]. Specifically, a mice model of experimental esophagitis induced by exposing to a respiratory allergen (Aspergillus fumigatus and dust mite mixture) was developed by Mishra et al. [49]. Mice exposed to the allergen revealed an increase in esophageal eosinophils, free eosinophil granules, and epithelial cell hyperplasia [49].

Atopy is present in 75% of patients with EoE [50]; in particular, it has been demonstrated that a history of atopic dermatitis (AD), IgE-mediated food allergy, and asthma are independently and cumulatively associated with the diagnosis of EoE [51]. In addition, children affected by IgE-mediated food allergy, especially those with multiple food allergies, can develop EoE at 9 times the rate of children without this disorder [52]. Similarly, the risk of EoE development in patients suffering from IgE-mediated food allergy who are on oral immunotherapy increases to 2.7–5.3% [53]. Additionally, an association between EoE, asthma and airway hyperresponsiveness (AHR) has been highlighted [54]: a cross-sectional prospective study demonstrated that AHR is more frequent in subjects affected by EoE than in the control group (33% vs. 11%) and a high level of s-IgE in these patients is associated with a greater risk of AHR.

Early-life environmental exposures play a role too: maternal fever, pre-and postnatal antibiotics, proton pomp inhibitor (PPI) therapy, and neonatal intensive care unit admission in early life are associated with an increased risk of EoE [26, 55]. The theory is that early exposure could alter gut microbiota, which in turn may alter the development of the normal immune system [56].

**Fig. 1:**
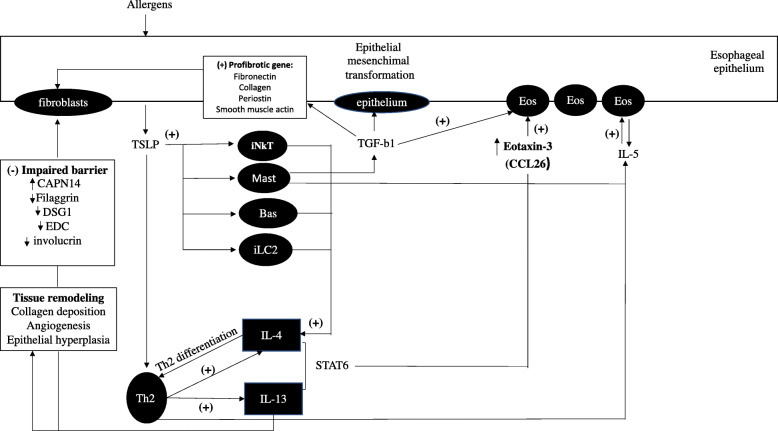
Overview of EoE pathogenesis (adapted from reference [59]). Bas, Basophils; Eos, Eosinophils; IL, Interleukin; iLC2, innate lymphoid cells type 2; iNKT, invariant Natural Killer T cells; Mast, mast cells; Th, T helper cells. Allergens stimulate the esophageal epithelium inducing TSLP, leading to stimulation of Th2 cells, NK cells, mast cells, basophils, and iLC2. These cells induce IL-4 which promotes Th2 differentiation. IL-4 and IL-13 induced by Th2 cells induce eotaxin-3 (CCL26), which stimulates eosinophils to secrete IL-5. IL-5, secreted by Th2 cells and mast cells, stimulate eosinophils as well. Mast cells also induce TGF-b1 which stimulate eosinophils and fibroblasts. Furthermore, IL-13 induces impaired barrier function and tissue remodeling

On the other hand, *Helicobacter pylori* (*H. pylori*) seems to have a protective effect on esophageal eosinophilia [57]. This evidence could be explained by the fact that *H. pylori* may drive a Th1 mediated allergic response, shifting the Th1–Th2 balance and protecting from an allergic Th2 response [58]. An overview of EoE pathogenesis is shown in Fig. 1.

## Clinical manifestations

**Fig. 2:**
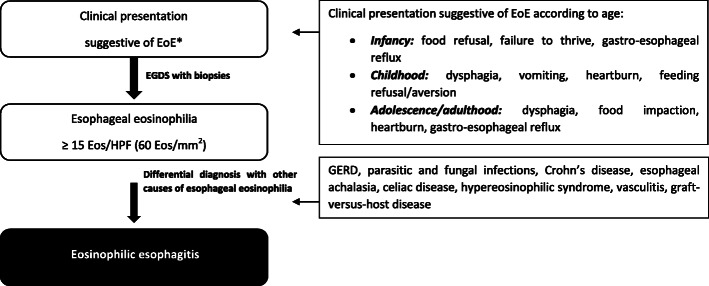
Clinical manifestation and diagnostic algorithm for eosinophilic esophagitis (Modified from reference [62]). EGD, esophagogastroduodenoscopy; EoE, Eosinophilic Esophagitis; Eos, Eosinophils; GERD, gastroesophageal reflux disease; HPF, high-power field

EoE is characterized by different clinical manifestations according to different ages of onset [60, 61]. Infants present with food refusal, failure to thrive and gastroesophageal reflux; school-aged children present with symptoms of gastroesophageal reflux and finally, adolescents and adults presents with dysphagia and food impaction. Clinical manifestation of EoE in infancy, childhood and adolescence/adulthood is indicated in Fig. 2.

These differences in clinical onset is postulated to derive from different causes: first of all, infants and children fail to express their symptoms in the same manner as adolescents and adults, secondly, the esophageal tissue remodeling is progressive and develops over time from an inflammatory phenotype in childhood to a fibrostenotic phenotype in adulthood [14, 63–65].

Moreover, the symptoms could be masked, because the patients may have adapted compensatory feeding habits (eating slowly, excessive mastication, cutting food into small pieces) and dietary changes (preference for liquids and soft food, and avoidance of specific textures like meat and bread) to prevent the development of symptoms (dysphagia and food impaction) [58, 60, 62, 66, 67].

Therefore, it is of paramount importance to include the following questions in the medical history: “Do you chew your food a lot?” “Are you the last to get up from the table?” “Do you drink plenty of water during the meal to help you swallow?” “Do you avoid eating certain foods such as bread, rice, or meat?” “Do you cut the food into very small pieces?” [60, 68].

Since EoE symptoms are unspecific, especially in infants and children, diagnosis is reached with an average delay of 3–5 years [69, 70], with an increasing risk of developing fibrosis [70]. Indeed, the risk of fibrostenosis increases by 5% with each year of symptoms before diagnosis [70].

Extraesophageal symptoms could be associated with EoE as reported for the first time by Orenstein et al. [71]. It is unclear whether the respiratory symptoms were caused by EoE or whether the relationship was due to allergic conditions coexisting in a cohort of children having a common atopic background [72]. There are a lot of theories on this subject: first of all, the hypothesis that explains the onset of respiratory symptoms due to the generating of proinflammatory cytokines by the eosinophils living in the upper esophagus [73–75]. Alternatively, other authors have hypothesized the role of micro-aspiration, resulting in food antigens gaining direct access to the immune system via the lungs while circumventing the gastrointestinal tract [71]. According to this hypothesis, the interaction of food antigens directly with the immune system causes the development of an allergic response and subsequently, EoE [71]. However, several studies are necessary to understand in detail the interrelationship between esophageal and respiratory symptoms in patients affected by EoE [72].

EoE is strongly associated with some comorbidities, such as atopic conditions, and therefore these patients should be screened routinely for EoE [51]. In particular, 26–50% of EoE patients have concomitant asthma, 30–90% have associated allergic rhinitis, 19–55% have atopic dermatitis and, 9.8–68% have IgE-mediated food allergy [52, 76–80]. Moreover, other non-atopic diseases have been shown to be associated with EoE, such as inflammatory bowel disease [81] connective tissue disorders [82], autism [83], attention deficit hyperactivity disorder [83], celiac disease [83, 84] and other monogenic disorders [83, 85]. This group of patients may be overrepresented in 21–25% cases by those who do not demonstrate concomitant allergic disease [78], as they could be represented by a different EoE-phenotype [86].

When EoE is suspected based on symptoms, the EGD with biopsy is the only way to confirm the diagnosis [1, 61].

## Diagnosis

The diagnosis of EoE has undergone several updates in the last two decades with the birth of new concepts and evidence [16, 69, 87, 88]. In 2018, an international consensus [1] based on a systematic review of literature and expert opinions revised the EoE diagnostic criteria. It defined EoE as a clinicopathologic disease characterized by [1] esophageal symptoms, e.g., dysphagia and food impaction in adults, and feeding intolerance and gastroesophageal reflux disease (GERD) symptoms in children, together with [2] eosinophil-predominant inflammation of 15 or more eosinophils per high-power field (hpf) (i.e., ≥15 eos/hpf) in the esophageal tissue [3] after exclusion of other disorders associated with similar clinical, histologic, or endoscopic features [1]. The diagnostic algorithm for EoE is illustrated in Fig. 2 [62].

The main novelty introduced by the recent consensus guidelines is the removal of failed twice-daily or high-dose proton pump inhibitor (PPI) therapy before diagnosing EoE. This derives from the evidence that although PPIs primarily provide acid blockade, they can also have anti-inflammatory effects (e.g., decrease of IL-13–induced eotaxin-3 production) [89, 90]. Currently, PPI-responsive esophageal eosinophilia (PPI-REE) is considered an EoE sub-phenotype, since it does not differ from EoE in clinical, endoscopic or histological characteristics and, therefore, PPI is considered a therapeutic option for EoE on the same level as diets and topical steroid treatment, and as such, is no longer a diagnostic criterion [1].

The gold standard for EoE diagnosis is still biopsy findings that demonstrate increased intraepithelial esophageal eosinophil counts without concomitant eosinophilic infiltration in the stomach or duodenum [16]. Since eosinophilic infiltration of the esophagus might be patchy (i.e., non-continuously distributed), at least five biopsy specimens should be obtained at multiple levels from the proximal and distal esophagus to maximize sensitivity, targeting mainly areas of apparent inflammation. Together with the key diagnostic criterion of esophageal eosinophilia (i.e., ≥15 eos/hpf), other histologic features of EoE include superficial layering of the eosinophils, eosinophilic micro-abscesses (clusters of > 4 eosinophils), basal zone hyperplasia, dilated intercellular spaces, surface epithelial alteration, dyskeratotic epithelial cells, and lamina propria fibrosis. Some patients with EoE, adults and children alike, may present subepithelial fibrosis in their biopsy specimens [91, 92]. Based on current knowledge, a newer EoE histologic severity scoring index has been developed, called the EoE histologic scoring system (EoEHSS) [93], which does not focus solely on eosinophil numbers but also takes the abovementioned histologic features into account [93].

Although the histologic feature of eosinophilia is the key factor for a diagnosis of EoE, it is not pathognomonic of EoE [1]. Therefore, to formulate a proper diagnosis, it is essential to investigate any secondary causes of esophageal eosinophilia. The differential diagnosis for EoE is broad and can include GERD, parasitic and fungal infections, Crohn’s disease, allergic vasculitis, connective tissue disease, and other disorders associated with esophageal eosinophilia [1]. For an accurate diagnosis and management, it is important to treat the potential primary disease and evaluate whether this induces the remission of esophageal eosinophilia. If still present, then a diagnosis of concurrent EoE is formulated. Often it is not initially possible to make a distinction between EoE and GERD as the symptoms of these 2 diseases overlap [94]. However, if symptoms fail to improve with PPIs or if they recur shortly after PPI suspension, an esophago-gastro-duodenoscopy (EGD) is recommended to detect eosinophilic infiltration. If more than 15 eos/hpf are found, the diagnosis of EoE is made even if there are typical reflux symptoms [1].

Furthermore, it is recommended to assess whether any iatrogenic EoE triggers (e.g., oral immunotherapy, OIT) exist. Specifically, a minority of patients (2.7 to 5.7%) undergoing OIT for the treatment of food induced anaphylaxis are at risk of EoE [53, 95]. If patients develop EoE during OIT, they are advised to discontinue the therapy, usually with an improvement in the clinical and histological EoE manifestations. Even though cases of sublingual immunotherapy inducing EoE have been reported, they are rare [96–98].

### Less invasive techniques to evaluate the esophagus

Researchers have focused on the development of less invasive tools to evaluate esophagus inflammation, since upper endoscopy is an invasive and expensive procedure, even requiring general anesthesia in children and conscious sedation in most adult patients. Moreover, in 2016, the Food and Drug Administration issued a safety warning states that “repeated or lengthy use of general anesthetic and sedation drugs during surgeries or procedures in children younger than 3 years or in pregnant women during their third trimester may affect the development of children’s brains”, caused increase concerns over anesthetic exposure at a young age leading to anesthetic-induced neurotoxicity [99, 100]. Since then, more caution has been used to do repeated EGD under anesthesia.

Un-sedated transnasal endoscopy has been used in children and adults to assess esophageal mucosal inflammation through biopsy [6].

Although radiography is not useful in identifying the inflammatory findings of edema and exudates, it can be helpful in evaluating fibrostenotic disease [7, 8]. Barium esophagography is more sensitive than endoscopy for detecting esophageal stricture and diffuse small-caliber esophagus [9].

An instrumental examination currently being validated is the esophageal string-test (EST). Consisting of a nylon thread the distal end of which is attached to a gelatin capsule that captures eosinophil-associated proteins from the esophageal lumen, it has shown good correlation with eosinophilic infiltration in esophageal biopsy specimens in both children and adults [10]. Another capsule-based technology is the Cytosponge (Medtronic, Minneapolis, Minn); originally designed for assessment of the esophageal mucosa in patients with Barrett’s esophagus, it has recently been used to assess inflammation in adult patients with EoE [11].

A tethered confocal microscopy capsule has been piloted in adults with EoE, with preliminary results suggesting that comprehensive cellular data can be gathered for assessing tissue inflammation [12]. All these methods are promising for assessing inflammation without the use of standard endoscopy. Although they are unlikely to replace the diagnostic or therapeutic benefits of endoscopy when a diagnosis or dilation is needed, they could play an important role in replacing repeated endoscopies for disease surveillance after treatment interventions.

Other non-invasive biomarkers are also under investigation for reducing the need for repeated endoscopies and biopsies which entail significant costs, risks, and discomfort for patients, [101]. Several candidates have been studied, mainly in the blood/serum, but also in the urine, stools and breath testing [101]. The composition of the salivary microbiome community structure has been shown to be altered in children with EoE [102, 103].

Fractionated exhaled nitric oxide testing (FeNO) is a standardized non-invasive test with proven utility in the evaluation of asthma. The use of elevated FeNO has also been reported in other eosinophilic inflammatory conditions. However, in eosinophilic esophagitis, FeNO has limited clinical efficacy in predicting severity of esophageal eosinophilia. A study conducted in 50 patients with EoE has shown that in patients with FeNO levels > 40 ppb, specificity of testing was high, but very few patients reached this FeNO level [104].

### Emerging diagnostic tools

Thickening of the deeper layers of the esophagus has been demonstrated via use of endoscopic ultrasonography [105, 106]. The endoscopic functional luminal imaging probe (endoFLIP) is a new technique for assessing mucosal and submucosal fibrosis and smooth-muscle hypertrophy which are likely to cause decreased esophageal compliance, contributing to dysphagia symptoms in the absence of an identifiable stricture [107]. A balloon mucosal impedance (MI) catheter system that instantly detects changes in esophageal mucosal integrity during endoscopy over a long segment of the esophagus during endoscopy has been found to be safe and reliable in identifying patients with GERD, EoE, or non-GERD [108]. Furthermore, endoscopic mucosal impedance measurements correlate with eosinophilia and dilation of intercellular spaces in patients with EoE, adults and children alike [11, 109].

While histologic assessment is the gold standard in diagnosing EoE, in those patients with a high pre-test probability of EoE and eosinophil count < 15 hpf, tissue staining for eosinophil products, such as eosinophil peroxidase (EPX), might be useful, even though EPX staining remains a research tool [110].

Furthermore, the eosinophilic esophagitis diagnostic panel (EDP) is a molecular tool which assesses the expression of 96 genes that are dysregulated in patients with EoE and has high sensitivity and specificity for diagnosis and molecular phenotyping in EoE [111]. In this regard, a multicenter cross-sectional study analyzed 185 esophageal biopsy specimens from pediatric and adult patients with EoE, using EDP. Histological and endoscopic features were assessed by quantification of esophageal eosinophils and via use of the eosinophilic esophagitis histology scoring system (EoEHSS) and the eosinophilic esophagitis endoscopic reference score (EREFS). In a validation cohort consisting of 100 specimens, the EDP identified three clusters significantly associated with distinct endotypes (termed EoEe1–3) despite similar eosinophil levels. EoEe1 was associated with a normal-appearing esophagus (risk ratio [RR] 3.7, 95% CI 1.04–10.27; *p* = 0.0443), an inverse association with a history of esophageal dilation (0.27, 0.09–0.82; *p* = 0.0105), and relatively mild histological, endoscopic, and molecular changes. EoEe2 showed an inflammatory and steroid-refractory phenotype (RR 2.77, 95% CI 1.11–6·95; *p* = 0.0376) and had the highest expression of inflammatory cytokines and steroid-responding genes. EoEe3, which was associated with a narrow-caliber esophagus (RR 7.98, 95% CI 1.84–34.64; *p* = 0.0013) and adult onset (2.22, 1.19–4.12; *p* = 0.0155), showed the highest degree of endoscopic and histological severity and the lowest expression of epithelial differentiation genes. It has been speculated that this classification might have potential clinical and therapeutic significance in the perspective of a precision-medicine approach to EoE [31].

## Therapy

The aims of EoE therapy include the accomplishment of clinical and histological remission in order to prevent long-lasting untreatable fibrosis [63–65].

**Fig. 3:**
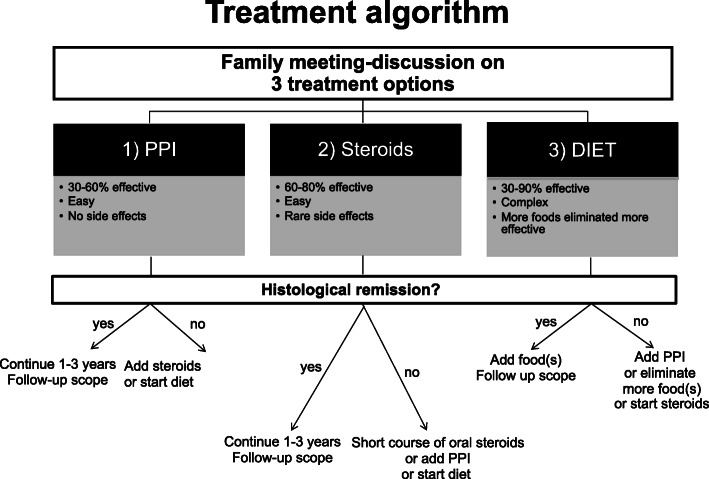
Clinical practice algorithm for management of pediatric eosinophilic esophagitis (Modified from reference [113–115]). PPI, proton-pump inhibitor

As the therapy is likely to be lifelong, the prevention of iatrogenic impairment triggered by adverse events of pharmacological treatments and long-standing elimination diets is crucial [112]. The clinical practice algorithm for the management of pediatric eosinophilic esophagitis is illustrated in Fig. 3.

### Acid suppression

According to the recent consensus guidelines [1], PPIs can be chosen as a first-line treatment option like steroids and elimination diets, since updated diagnostic criteria [1, 113] indicate that a failed PPI trial prior to endoscopy is not necessary for making a diagnosis of EoE. Given the simplicity of administration and lack of significant side effects, they are often the first choice [69, 116, 117], especially among patients with milder symptoms, low inflammation, and low levels of fibrosis [111]. They are used at high dosages (e.g., 40-mg omeprazole is administered twice a day or 1–2 mg/kg in children), probably to exploit their anti-inflammatory properties [89, 90]. An EGD needs to be repeated to prove its efficacy as often symptoms that improve with PPI treatment may still be associated with profibrotic esophageal inflammation requiring a step-up treatment [116]. PPI are considered safe drug, nevertheless possible recognized side effects of long-term acid suppression are dysbiosis, malabsorption, osteoporosis, and possible higher risk for gastrointestinal and respiratory infections [118, 119].

The first-line options for pediatric EoE treatment are summarized in Table 1.

**Table 1:** First-line therapies in pediatric eosinophilic esophagitis

Drug	Daily induction dosing (usually divided doses)^**c**^
***Proton pump inhibitors***	
Omeprazole	1 mg/kg BID (max 20–40 mg)
Lansoprazole	1 mg/kg BID (max 60 mg)
Esomeprazole	1 mg/kg QD (max 40 mg)
Pantoprazole	< 10 years of age: 1 mg/kg QD (max 40 mg) > 10 years of age: 20 mg QD (max 40 mg)
***Topical steroids***	
Swallowed, inhaled fluticasone propionate^a^	88–440 μg BID-QID (max 880–1760 μg)
Oral viscous budesonide^b^	< 10 years of age: 0.5 mg BID (max 4 mg) > 10 years of age: 1 mg BID (max 4 mg)
***Systemic steroids***	
Prednisone	1–2 mg/kg BID (max 30 mg BID)

^a^ the patients should be instructed to puff the medication in the mouth during a breath-hold and not drink, eat or wash mouth for at least 30 min after swallowing

^b^ Budesonide orodispersible tablets are available in several European countries for adults only.

^c^ Specific doses in children will be determined by age, height or weight.

Legend: *BID* Bis in die, two times a day, *QD* Quoque die, once a day, *QID* Quater in die, four times a day; max: maximum.

### Dietary modifications

Elimination of allergens and antigens from diet addresses the removal of the causes of inflammation and should consider the patients’ lifestyle, adherence aptitude, and family resources [67, 120, 121]. While popular a few years ago, they are now used less frequently as more pharmacological options become available [121]. Several dietary strategies have been developed: 1) the targeted elimination diet (TED), based on allergy testing in order to recognize potential allergens and eliminate them from the diet; 2) the empiric elimination diet (EED) or six-food elimination diet (SFED) that removes the most frequent foods causing allergic reactions (e.g. the “big six”: cow’s milk, egg, soy, wheat, peanuts/tree nuts, and fish/shellfish); 3) the elemental diet (ED) that avoids all potential allergenic food proteins altogether. After the establishment of a histologic disease activity resolution, excluded foods should be reintroduced separately to achieve a less restrictive and more effective diet. Elimination diets suggest an overall success rate that varies between 45 and 90% depending on the different approaches. In particular, the ED resulted in a histologic response of approximately 90% in meta-analysis, but with high patient nonadherence and dropout because of low palatability [122–124]. According to the recent guidelines [1], an ED should only be used for 4 weeks as a last resort in highly refractory cases followed by an EGD to show resolution, and the fast reintroduction of foods should be recommended [121]. In fact, while ED can be useful and has an efficacy rate of at least 90% in children affected by multiple food allergies, growth deficiency, refractory disease, and highly unbalanced diets, in older children it almost always requires nasogastric tube feeding due to its unpleasant taste and it can mask a feeding disorder, especially in malnourished patients [125–127].

Empiric elimination in these diets can be performed with a step-up approach, starting with the elimination of the most common triggers (1 or 2 foods, such as wheat and milk) and progressively excluding other foods until histologic resolution is achieved with an endoscopy. Otherwise, a step-down approach can be applied, starting with a highly restrictive diet (the most commonly used is SFED) and progressing with gradual food introduction. Although SFED has been well analyzed in the treatment of EoE, with histologic remission in 72% of children and 65–70% with a 1–2 food elimination diet, the latter seem to have better long-term compliance with fewer endoscopies so it is generally preferred [121, 122, 128, 129]. Endoscopy is performed 6–12 weeks after dietary variations to evaluate histologic disease activity. TD has been less popular in the last few years since IgE testing does not predict food allergy triggers in EoE and path testing is scarcely specific or sensitive and it is not standardized. Indeed, the success rate of TD is lower than SFED [50, 121, 130].

The different elimination diets are summarized in Table 2.

**Table 2:** Characteristics of dietary approaches in the treatment of pediatric eosinophilic esophagitis

Dietary approach	Definition	Indication	Success rate	Advantages	Disadvantages
Elemental diet	Diet consisting of amino acid-based formula	In patients with multiple allergies, growth stop, severe disease unresponsive to therapy or unable to follow a highly restrictive diet	90%	Allergen-free Nutritionally complete	Taste (feeding tube could be needed) Expensive Age relevance Elimination of all foods Negative impact on the quality of life
Empiric elimination diet or six-food elimination diet	Elimination of “big six” major food allergens from the diet (milk, egg, wheat, soy, peanut/tree nut, and fish/shellfish)	In the absence of specific allergic sensitization to foods	72%	Allergy testing not needed	Several eliminations could be unnecessary Only four foods may be essential Expensive Nutritional deficiency
Targeted diet	Elimination of foods with a positive response to allergy testing	Strongly suspected food allergy based on the clinical history and positive allergy testing	45–77%	Food specificity Nutritional preservation	Different testing precision and technique among centers Low negative predictive value of milk testing Unnecessary avoidance if sensitization without clinical allergy

The major detriments of dietary elimination in children are nutritional deficiency, decreased quality of life, psychological impact and the risk of developing feeding disorders (e.g anorexia and bulimia, especially in adolescents) [112].

### Steroids

Due to being highly successful in patients with EoE, topical corticosteroids are often used as first-line treatment in more severe cases or in those that fail with PPIs. They induce clinical and histological remission, consistently prevent fibrosis and require fewer endoscopies, hence they are often preferred over diet treatment. While systemic steroids are highly effective in inducing quick remission in EoE and may be useful in the treatment of patients with fibrosis and severe symptoms (severe dysphagia, dehydration, weight loss, or esophageal strictures), they are hampered by several known adverse effects (e.g. weight gain, cushingoid manifestations), therefore the benefit-cost ratio should be discussed with the patient’s family [131]. The most common topical steroids are represented by swallowed fluticasone propionate and oral viscous budesonide, which is an off-label use of anti-asthmatic medication [132, 133]. It is important to underline that patient should fast at least 30–60 min after treatment to reduce esophageal drug clearance. Budesonide orodispersible capsules approved for EoE are available for adults in some European countries (https://www.ema.europa.eu/en/documents/assessment-report/jorveza-epar-public-assessment-report_en.pdf). In general, swallowed topical corticosteroids only have a few adverse effects, like esophageal candidiasis that is mostly asymptomatic and discovered during the endoscopy phase [116, 134]. Fluconazole maybe used for treatment [116].

Swallowed steroids are used for long-term treatment, often indefinitely in the case of efficacy in controlling remission 8–12 weeks after initiating therapy. Attempts to reduce dosage may be made after 1–2 years of treatment followed by an endoscopy to demonstrate efficacy. Periodic rescopes are recommended even after remission as at times patients may become non-responsive to steroids [126].

Although adrenal insufficiency could be a side effect due to the use of prolonged high dose of steroids, the risk is negligible in patients with short-term topical corticosteroids (< 12 weeks) [135]. More attention to the adrenal axis should be considered in patients with concomitant use of additional topic steroids for comorbid allergic diseases (e.g asthma, atopic dermatitis, allergic rhinitis) [135, 136].

No evidence is now available regarding the potential role of topical steroids on growth impairment in patients with EoE [62]. In patients affected by asthma, a systematic review showed that regular use of inhaled corticosteroids (ICS) at low or medium daily dose is associated with reduction on linear growth that seems to be maximal during the first year of therapy and less pronounced behind the first year of treatment [137]. For that reason, as well as it is suggested to use the lowest effective dose of ICS in asthmatic patients, in the same way it is advisable for patients with EoE.

The different types of steroids are summarized in Table 1.

### Endoscopic therapy

Endoscopic esophageal dilatation may be effective in case of rings or high-grade strictures (less than 10 mm), or for the release of the bolus, especially when severe dysphagia is caused [138]. This approach which treats the fibrostenotic and structural alterations, shows good tolerance with long-lasting symptomatic relief. It should be considered in cases that are unresponsive to initial medical or diet therapy. Efforts are made to achieve an esophageal diameter of 16–17 mm aimed at preventing and treating food impactions. Although very infrequent, surgical esophagectomy is the extreme treatment option when complications occur.

### Other therapies

Other drug treatments have been studied, such as leukotriene receptor antagonists (montelukast) [139], biologic agents (e.g., omalizumab, infliximab, mepolizumab, reslizumab) [140–144], immunomodulators (e.g., azathioprine, 6-mercaptopurine) [145] and oral viscous sodium cromoglicate [146], but without showing efficacy.

More promising are the data on biologics like dupilumab in treating EoE. Data from the first phase III trial of dupilumab, an antibody that inhibits the signaling of Il-4 and IL-13 proteins, highlighted how this drug changes the structural and histologic characters of EoE and decreases the patients’ symptoms, such as the ability to swallow [147]. Hence, in September 2020, the FDA approved breakthrough investigational therapy with dupilumab for treating EoE in patients aged 12 years and older (https://www.globenewswire.com/news-release/2020/09/14/2092666/0/en/FDA-grants-Dupixent-dupilumab-Breakthrough-Therapy-designation-for-eosinophilic-esophagitis.html), although it use in clinical practice has not yet been approved. Many other biologics are currently being studied for this disease [62].

## Conclusion

Over recent decades, research progress has been made in terms of a greater understanding of the EoE pathogenesis, evaluation of less invasive diagnostic tools, and new therapeutic approaches.

However, there are still several unmet needs (Table 3), such as finding non-invasive disease-monitoring methods and biomarkers for daily practice and the development of safe long-term maintenance therapy. Moreover, multidisciplinary management of EoE is necessary, involving pediatricians, gastroenterologists, allergists, pathologists, and dietitians for the optimization of patient care.

**Table 3:** Unmet needs in the management of pediatric eosinophilic esophagitis (modified from reference [62])

Diagnosis	Identify diagnostic and monitoring noninvasive biomarkers
	Increase the development of minimally invasive tools to acquire esophageal tissue
	Validate score to predict disease activity
Diet therapy	Cross-reactivity between foods and airborne allergens
	The timeframe of reintroduction: 6 versus 8 versus 12 weeks
	Predictive factors of food-elimination responsiveness
	Long-term response in adherent patients
Drug maintenance therapy	Dose and persistence of maintenance treatment (PPI and steroids)
	Safety of long-term minimum effective dose
	The predictive factor of steroid response and dependence
Other therapy	Validation of current available biologic agents
	Development of new agents targeting identified molecules
	Identification of possible new targets for biologic therapy

Legend: *PPI* proton-pump inhibitor
